# Adolescent with cannabis use disorder and other mental health disorders: does dual disorder worsen school impairment?

**DOI:** 10.1186/s42238-026-00449-1

**Published:** 2026-05-26

**Authors:** J. L. Matali, E. Flores, X. Estrada-Prat, A. Bonillo

**Affiliations:** 1 Department of Child and Adolescent Mental Health, Hospital Sant Joan de Déu, Barcelona, Spain; 2 Child and Adolescent Mental Health Research Group, Institut de Recerca Sant Joan de Déu, Barcelona, Spain; 3 Centro de Investigación Biomédica en Red de Salud Mental (CIBERSAM), Madrid, Spain; 4 Department of Psychobiology and Methodology in Health Sciences, Autonomous University of Barcelona (UAB), Bellaterra, Spain

**Keywords:** Adolescents, Dual diagnosis, School impairment, Cannabis

## Abstract

**Introduction:**

Despite evidence of the impact on academic performance of cannabis use disorder (CUD) there is little information about what happens in those cases of dual diagnosis, when CUD occurs along with other mental health disorders (DD).

**Methods:**

Participants in this study (*N* = 300) were recruited from patients seeking treatment for CUD at an adolescent addictive behaviors unit. A multivariate logistic regression analysis was conducted to evaluate the effects of DD on school impairment (listwise deletion strategy). The interaction between CUD and other mental health disorders was included, along with covariates such as age of onset, years elapsed from first use to problematic use, and sex.

**Results:**

The presence of co-occurring internalizing and/or externalizing disorders among adolescents with CUD was associated with higher odds of school impairment (OR = 3.73; 95% CI: 1.06–13.12; *p* = .04). In the adjusted model, psychiatric comorbidity was also associated with approximately twice the likelihood of repeating a grade or dropping out of school (OR = 2.05; 95% CI: 1.10–3.84; *p* = .03), whereas the CUD-only group, sex, age of initiation, and latency to regular cannabis use were not statistically significant predictors. These findings suggest that school impairment is particularly associated with the co-occurrence of CUD and other mental health disorders.

**Conclusions:**

DD in adolescents emerges as a risk factor for academic performance. It is crucial to develop early intervention policies and approaches that comprehensively address DD.

## Introduction

Cannabis is the most widely used illicit drug globally and the most prevalent among adolescents, with boys being greater consumers than girls (UNODC WDR 2024 (United N publication, 2024) 2024). Cannabis use is associated with multiple long-term consequences, one of the most significant being the impact on academic outcomes (Chan et al. 2024). These consequences are particularly critical among those individuals who begin cannabis use at an early age (between 11 and 14 years old) (Halladay et al. 2024). From a cognitive perspective, early cannabis use has been linked to a greater impairment of visuospatial and executive functioning, attention, language, and abstract reasoning, as well as delayed memory (Bassiony et al. 2021). This cognitive impairment is associated with a lower high school graduation rate (Burke et al. 2021) and poorer educational and occupational achievement in adulthood (Zuckermann et al. 2020). Recent longitudinal and neurodevelopmental research further suggests that early and more intensive cannabis exposure may interfere with brain maturation processes relevant to executive functioning and learning, thereby increasing vulnerability to academic difficulties (Albaugh et al. 2021).

Mental health in adolescents and academic performance are intricately related (Lee et al. 2024). Academic performance is a significant predictor of mental health outcomes in adulthood (Monzonís-Carda et al. 2024) and the presence of a mental health disorder during childhood and adolescence is linked to poorer school performance (Crespo Molero and Sánchez Romero 2019) increasing the risk of absenteeism and lower educational attainment (Dalsgaard et al. 2020). Additionally, adolescents with poor academic performance, exposure to psychosocial difficulties or negative stressors, as well as those with a mental health disorder, are at increased risk of developing substance use–related problems (Connor et al. 2021). This relationship is likely bidirectional, as mental health difficulties may impair academic functioning, while school failure and disengagement may, in turn, exacerbate psychological distress and increase vulnerability to substance use.

What happens to academic performance when a mental health disorder coexists with cannabis use disorder (CUD), a condition commonly referred to as dual diagnosis (DD)? DD is defined as the co-occurrence of cannabis use disorder (CUD) and at least one additional mental disorder. This question is particularly relevant given that co-occurring CUD and mental health comorbidities represent a major clinical challenge in adolescent treatment settings (MacPherson et al. 2021; Lees et al. 2021). Previous studies conducted in adolescent clinical and treatment settings have reported DD prevalence rates ranging from approximately 60% to 80%, particularly among youths receiving specialized mental health or addiction care (Jandac and Stastna 2024). The most prevalent diagnoses among adolescents with CUD are behavior disorders, followed by anxiety and depressive disorders (Jandac and Stastna 2024). The presence of a DD has been associated with greater severity of mental health symptoms and a worse prognosis (Halladay et al. 2020). Psychiatric disorders in adolescence are commonly organized into internalizing and externalizing domains, consistent with established psychopathological taxonomies and supported by hierarchical models of psychopathology (Castellanos-Ryan et al. 2016). Compared to patients with a single disorder, whether it is a substance use disorder or another psychiatric condition, those with DD exhibit greater psychopathological severity, increased use of emergency services, higher rates of psychiatric hospitalization, greater prevalence of suicide, more interpersonal difficulties, and poorer treatment outcomes (Otasowie 2021; Adan and Torrens 2021).

These adverse clinical features may plausibly translate into poorer academic functioning through multiple mechanisms, including emotional dysregulation, cognitive impairment, behavioral problems, reduced concentration, and increased school absenteeism or disengagement (Becker et al. 2022). From a clinical and public health perspective, this highlights the importance of early identification of dual diagnosis and supports the need for integrated screening and intervention strategies across educational and mental health services (Volkow and Wargo 2022).

The existing literature provides a robust foundation for understanding how the presence of a mental health disorder negatively affects adolescents’ academic performance, highlighting the importance of early intervention (Meruelo et al. 2020). Similarly, a growing body of evidence has linked cannabis use to adverse educational outcomes (Burke et al. 2021). However, most studies have examined these factors independently, and relatively little is known about their combined or interactive effects (Halladay et al. 2024). Despite the existing evidence and the high prevalence of DD in clinical samples of adolescents, the extent to which dual diagnosis amplifies school impairment in adolescents with cannabis use disorder remains insufficiently understood.

The present study aimed to examine the association between dual diagnosis and school impairment in adolescents with cannabis use disorder, as well as the role of cannabis-related clinical variables (age of onset and latency to regular use). We further examined whether dual diagnosis modifies these associations. We hypothesized that dual diagnosis would be associated with higher odds of school impairment, that earlier onset and shorter latency would predict worse academic outcomes, and that dual diagnosis would amplify these associations.

## Method

Initial participants (*N* = 360) were recruited between 2019 and 2025 from patients seeking treatment for CUD at an outpatient adolescent addictive behaviors unit at Hospital Sant Joan de Déu (Barcelona, Spain). The study was conducted in a tertiary care center serving a predominantly urban catchment area in Barcelona. Participants were primarily referred from child and adolescent mental health services. This was a prospective cohort study with a 6-month follow-up. Although the broader clinical cohort included 6-month follow-up, the present analysis focused on baseline/clinical assessment data and school impairment status recorded during the initial evaluation.

The final analytical sample included 300 participants after applying a listwise deletion strategy, as approximately 17% of cases had missing values in at least one core study variable. An exploratory analysis of missing cases did not reveal significant associations with the main study variables (*n* = 300). Listwise deletion provides unbiased estimates primarily under a missing completely at random (MCAR) assumption. As this clinical study focused primarily on treatment delivery rather than a priori research recruitment, no formal sample size or power calculation was performed before data collection.

Inclusion criteria were being aged between 12 and 18 years (12–18 years; M = 15.49, SD = 1.18), fulfilling DSM-5 criteria for CUD, assessed with the K-SADS-PL-DSM5. Participants were subsequently classified according to the presence or absence of additional mental disorders. Exclusion criteria were the presence of any acute mental or somatic health condition that prevented the subject from understanding the questionnaire items. No participants meeting inclusion criteria were excluded after eligibility assessment. Detailed information regarding the number of patients approached, excluded, or declining participation was not systematically recorded. However, participants were recruited consecutively within a clinical setting, which may reduce the risk of selection bias. Information on participants’ ethnic background was not systematically collected as part of the clinical assessment and is therefore not available.

This study was approved by the Ethics Committee of the Sant Joan de Déu Hospital (N 001UCAD). All participants were informed about the study, read the confidentiality agreement, and consented to be involved. Informed consent was obtained from parents or legal guardians, and assent was obtained from all adolescents prior to participation.

CUD and other mental health diagnoses were assessed with the Kiddie-Schedule for Affective Disorders and Schizophrenia Present and Lifetime-DSM-V (KSADS-PL-DSM-5), Spanish version (Peña et al. 2018). Assessments were conducted by trained clinical psychologists and psychiatrists based on information obtained from both adolescents and their caregivers. Diagnostic assessments were conducted as part of routine clinical practice. Clinicians conducting psychiatric assessments had access to general clinical information, including school attendance records; however, specific academic outcome data (grade repetition or dropout status) were collected separately and were not systematically used to inform diagnostic classification. Formal blinding procedures were not implemented, which represents a potential source of information bias. In our study, adolescents were classified as likely to have an internalizing disorder if they met criteria for any affective or anxiety disorder. On the other hand, participants were classified as likely to have an externalizing disorder if they met criteria for attention deficit hyperactivity disorder, behavior disorder, or oppositional defiant disorder (Gattamorta et al. 2017). Comorbidity was categorized into four groups: internalizing disorders, including depressive disorders and anxiety disorders; externalizing disorders, including attention-deficit/hyperactivity disorder, oppositional defiant disorder, and conduct disorder; mixed disorders, defined as co-occurring internalizing and externalizing disorders; and cannabis-only, defined as cannabis use disorder without additional psychiatric comorbidity.

Additional information about cannabis use included age of onset, age of onset of regular cannabis use, and the pattern of cannabis use during weekdays. Age of onset was defined as the age at first cannabis use. Age of regular use referred to the age at which participants transitioned to regular consumption, defined as cannabis use at least 1 time per week for a minimum period of one month. This pattern was measured by asking participants about cannabis use habits before going to school, during school hours, and after school. Finally, all patients and their caregivers were asked whether they were attending school, if they had repeated any school year, or if they had dropped out of compulsory education before completing it.

Educational information was obtained from both adolescent and caregiver reports. We used two levels of educational consequences in this study: (0) appropriate educational achievement (being in the age-appropriate grade/year), or (1) having repeated at least one year or dropping out of school. Although grade repetition and school dropout may represent different severity levels of academic impairment, they were combined into a single binary outcome due to the limited prevalence of dropout alone and to capture the broader construct of school failure as defined within the Spanish educational system. In Spain, compulsory secondary education (Educación Secundaria Obligatoria, ESO) consists of four academic years and is typically completed between the ages of 15 and 16. Grade repetition is a commonly used indicator of academic difficulty within this educational system.

### Statistical analysis

Analyses were conducted using IBM SPSS Statistics version 29 and statistical significance was set at α = 0.05, using two-tailed tests. Logistic regression analyses were conducted on the complete-case sample after listwise deletion of participants with missing values in any core model variable. Associations between psychiatric comorbidity and school impairment among adolescents with CUD were tested within logistic regression models and interpreted according to their statistical significance and direction. Relevant confounders—sex, age of initiation, and latency between first cannabis use and problematic use- were retained in the adjusted models independently of their statistical significance, following standard epidemiological recommendations, and interaction effects were evaluated through multiplicative terms in the logistic regression models (Hosmer et al. 2013).

## Results and discussion

Table 1 presents the main sample characteristics and cannabis use patterns, including sociodemographic variables and academic outcomes, which are further disaggregated into dropout and repetition, as well as consumption behaviors. All variables were comparable across sex, with no statistically significant differences observed; detailed sex-stratified data are not shown. For categorical variables, chi-square values ranged from χ^2^ = 0.01 to χ^2^ = 1.17, with corresponding *p*-values between 0.921 and 0.279. For continuous variables, F-statistics ranged from F = 0.26 to F = 1.12, with *p*-values between 0.608 and 0.291.

**Table 1:** Sample characteristics and cannabis use patterns (*N* = 300)

Variable	n (%)	Mean (SD)
Male sex	178 (58.7%)	
Dual diagnosis (DD)	248 (82.7%)	
Academic failure (grade repetition or dropout)	183 (61.7%)	
Dropout	82 (27.3%)	
Repetition	173 (57.7%)	
Cannabis use before school (Yes)	173 (63.4%)	
Cannabis use during school (Yes)	121 (44.3%)	
Cannabis use after school (Yes)	158 (57.9%)	
Age of initiation (years)		13.14 (1.45)
Age of regular use onset (years)		13.80 (1.53)

Information on cannabis use was collected, including age of initiation (M = 13.14, SD = 1.45) and age of regular use onset (M = 13.80, SD = 1.53), as well as weekday consumption patterns. This pattern was measured by asking participants about their consumption habits before attending school (Yes = 173, 63.4%), during school hours (Yes = 121, 44.3%), and after school (Yes = 158, 57.9%). Most participants were male (*n* = 178, 58.7%) and had a dual diagnosis (*n* = 248, 82.7%).

Regarding educational outcomes, 61.7% of participants (*n* = 183) showed school impairment, defined as either grade repetition or school dropout. Specifically, 57.7% (*n* = 173) had repeated at least one academic year, while 27.3% (*n* = 82) had dropped out of school. To avoid multicollinearity issues, we included the time between initiation and regular cannabis use in the model (latency, in years).

Of note, the logistic regression was conducted on the complete-case sample of *n* = 300 participants, following the application of listwise deletion to the initial sample (N ≈ 360). Approximately 17% of cases had at least one missing value across core variables. As noted in the Statistical Analysis section, non-random missingness cannot be formally excluded, which may have introduced some bias and should be considered when interpreting the results.

A multivariate logistic regression analysis was performed to determine the association between dual diagnosis and school impairment among adolescents with CUD. Psychiatric comorbidity status was included in the model, along with age of initiation, latency from first cannabis use to regular cannabis use, and sex as covariates. The Hosmer & Lemeshow test (χ^2^ = 8.94; df = 8, *p* = 0.348) showed acceptable model fit (Nagelkerke pseudo *R*^2^ = 8.7%).

A significant association was found between co-occurring internalizing and/or externalizing disorders and school impairment among adolescents with CUD (OR: 3.73; 95% CI: [1.06–13.12]; *p* = 0.04). Regarding the main effects, psychiatric comorbidity was associated with approximately twice the likelihood of repeating one grade or dropping out (OR: 2.05; 95% CI: [1.10–3.84]; *p* = 0.03), whereas the CUD-only group was not independently associated with higher odds of school impairment. Sex, age of initiation, and latency to regular cannabis use were not statistically significant predictors in the adjusted model. Therefore, these variables should not be interpreted as independent protective or risk factors in the present analysis.

**Fig. 1:**
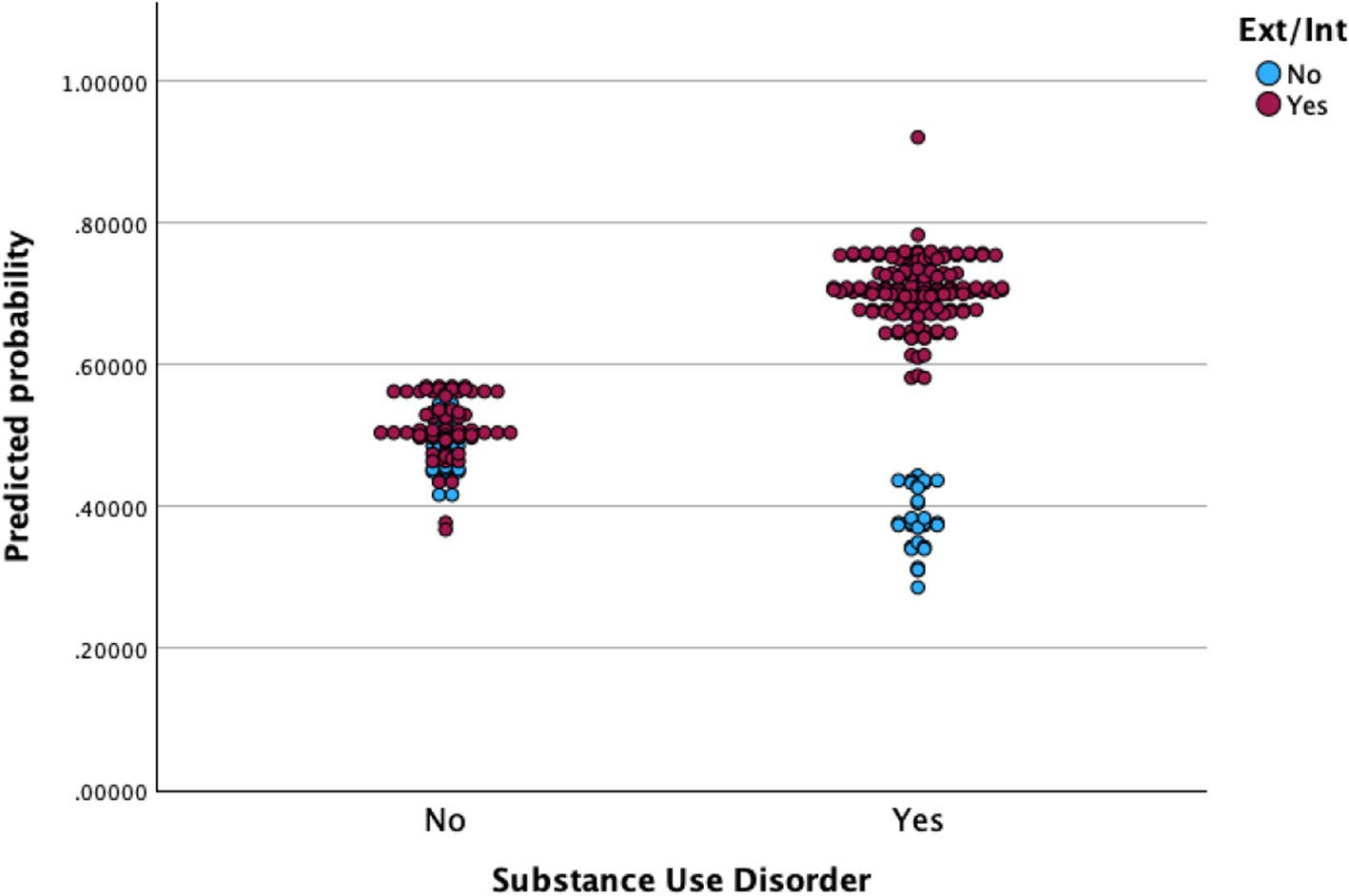
Model-adjusted predicted probability of school impairment according to comorbidity status. (*n* = 300)

Although female sex showed a higher point estimate, and older age of initiation and longer latency showed lower point estimates, none of these associations reached statistical significance. Consequently, the percentage changes derived from these odds ratios are presented only descriptively and should not be interpreted as statistically supported effects. Figure 1 summarizes the results of the model. Each point represents a single participant.

Predicted probabilities for academic failure for each person (point) were derived from the multivariable logistic regression model, with their values of age of initiation, latency (years from first use to problematic use), sex, cannabis use disorder and externalizing (Ext) and internalizing (Int) disorders.

The main finding of this study is that adolescents with DD showed poorer school performance. Specifically, co-occurring internalizing and/or externalizing disorders among adolescents with CUD were associated with higher odds of school impairment, suggesting that the combined presence of cannabis use disorder and psychiatric comorbidity may be particularly relevant for academic functioning. The proportion of school dropout or grade repetition was higher among adolescents with CUD and co-occurring psychiatric disorders than among those with CUD only, supporting the clinical relevance of considering psychiatric comorbidity when evaluating school impairment in adolescents with cannabis use disorder.

Previous studies have reported a relationship between early onset of cannabis use and lower high school graduation rates (Burke et al. 2021). Although early cannabis use has also been associated with poorer educational and socioeconomic outcomes in adulthood (Chan et al. 2024), age of initiation and latency to regular use were not statistically significant predictors of school impairment in the adjusted model of the present study. Therefore, these variables should be interpreted as clinically relevant background factors, but not as independent predictors of school impairment in our data.

When considering sex-related differences, female sex showed a higher point estimate in the adjusted model; however, this association did not reach statistical significance. Therefore, the present findings do not provide empirical support for sex differences in school impairment within this sample. Previous studies have reported sex-related differences in treatment trajectories, internalizing symptoms, cannabis-related motives, craving, and psychosocial outcomes among individuals with substance use disorders (Dahlberg et al. 2022; Foster et al. 2016; Gex et al. 2023; Fonseca et al. 2021; Tang and George 2024). In particular, adolescent girls with substance use disorders may present specific vulnerability profiles, including greater internalizing symptom burden, anxiety and depressive symptoms, suicide risk, and coping-related cannabis use motives (Foster et al. 2016; Gex et al. 2023). However, these mechanisms should be interpreted only as contextual hypotheses derived from previous literature and were not directly confirmed in the present study.

Another result of our study is that DD is associated with a higher likelihood of school impairment, suggesting that the presence of a DD is associated with poorer outcomes including lower academic performance (Adan and Torrens 2021). The bidirectional relationship between mental health and academic performance during adolescence (Lee et al. 2024) together with the importance of academic performance as a predictor of health in adulthood (Monzonís-Carda et al. 2024), highlight the importance of early interventions in adolescents with DD to improve their quality of life (Volkow and Wargo 2022).

Several limitations should be considered. First, the sample was predominantly male, which may limit the precision of sex-specific analyses and the generalizability of findings to female adolescents. Second, approximately 17% of the initial participants had at least one missing value among those used in the model (sex, age of initiation, years from problematic use, CUD, other diagnoses, and academic performance). The complete-case analysis was conducted under the assumption of missing completely at random (MCAR); however, missing at random (MAR) or missing not at random (MNAR) mechanisms cannot be formally excluded, and non-random missingness may have introduced bias in the estimates (Sterne et al. 2009). Third, the absence of a control group limits the ability to compare adolescents with CUD and DD with community adolescents or adolescents with psychiatric disorders but no cannabis use disorder. Fourth, clinical samples may differ from community samples in psychiatric severity, comorbidity burden, functional impairment, cannabis use patterns, and help-seeking pathways; therefore, caution is warranted when generalizing these findings to the broader adolescent population. Fifth, the study was conducted in an urban catchment area in Barcelona characterized by social, cultural, and ethnic diversity, but ethnicity was not systematically collected or analyzed. This limits our ability to examine whether cannabis use patterns, academic trajectories, or access to mental health services differed across ethnic or sociocultural groups. Sixth, the interpretation of school outcomes should be contextualized within the Spanish compulsory secondary education system (Educación Secundaria Obligatoria, ESO) and local educational and social support structures, which may differ substantially from those in other countries. Additional limitations include the absence of formal inter-rater reliability analyses for diagnostic assessments, the absence of detailed participant flow data, the lack of formal blinding of clinicians to academic outcome data during psychiatric assessments, and the observational clinical design, which limits the ability to establish causal relationships. Future research should include larger and more balanced samples, particularly with more female participants, longitudinal analyses of academic trajectories, and more detailed sociocultural and educational variables.

The present findings should be interpreted within the broader literature examining the relationship between adolescent mental health, substance use, and educational outcomes. Previous research has consistently shown that both substance use and psychiatric disorders are independently associated with poorer academic performance, including increased risk of school dropout, lower academic achievement, and functional impairment (Lee et al. 2024). However, relatively few studies have examined the combined impact of co-occurring psychiatric disorders and substance use on educational trajectories.

In this context, our results extend existing evidence by focusing on dual diagnosis and by examining the association between psychiatric comorbidity and school impairment among adolescents with cannabis use disorder. The findings suggest that their co-occurrence is associated with a substantially higher likelihood of school impairment compared to the presence of either condition alone, supporting the need for an integrated perspective on adolescent risk. Rather than reflecting additive effects only, this pattern may indicate a more complex vulnerability profile in which multiple clinical dimensions converge to affect academic functioning.

The results of the present study suggest that dual diagnosis, particularly the co-occurrence of CUD with internalizing and/or externalizing disorders, is associated with a higher likelihood of school impairment in adolescents. By contrast, sex, age of initiation, and latency to regular cannabis use were not statistically significant predictors in the adjusted model and should therefore be interpreted cautiously. These findings highlight the importance of early identification strategies targeting adolescents with cannabis use disorder and co-occurring internalizing and externalizing symptoms, particularly those showing broader clinical and functional vulnerability.

From a clinical and public health perspective, these results support the need for coordinated and integrated approaches across school systems, child and adolescent mental health services, and addiction services. Schools may play a key role in early detection through the identification of academic decline and behavioral changes, while mental health and addiction services may contribute to comprehensive assessment and intervention. Integrated care models that address both substance use and co-occurring psychiatric symptoms may be particularly relevant for adolescents with dual diagnosis, especially those showing broader clinical and functional vulnerability.

## Data Availability

The datasets used and/or analyzed during the current study are available from the corresponding author on reasonable request.

## Abbreviations

CUD: Cannabis use disorder
DD: Dual diagnosis
